# Advancing Lung Cancer Treatment with Combined c-Met Promoter-Driven Oncolytic Adenovirus and Rapamycin

**DOI:** 10.3390/cells13181597

**Published:** 2024-09-23

**Authors:** Shih-Yao Chen, Chung-Teng Wang, Tang-Hsiu Huang, Jeng-Liang Tsai, Hao-Tien Wang, Yi-Ting Yen, Yau-Lin Tseng, Chao-Liang Wu, Jia-Ming Chang, Ai-Li Shiau

**Affiliations:** 1 Department of Nursing, College of Nursing, Chung Hwa University of Medical Technology, Tainan 71703, Taiwan; leonlai50@gmail.com; 2 Tong Yuan Diabetes Center, College of Medicine, National Cheng Kung University, Tainan 70101, Taiwan; knight790105@hotmail.com (C.-T.W.); wumolbio@mail.ncku.edu.tw (C.-L.W.); 3 Department of Microbiology and Immunology, College of Medicine, National Cheng Kung University, Tainan 70101, Taiwan; 4 Division of Chest Medicine, Department of Internal Medicine, National Cheng Kung University Hospital, College of Medicine, National Cheng Kung University, Tainan 70101, Taiwan; tangomycin0713@gmail.com; 5 Department of Biochemistry and Molecular Biology, College of Medicine, National Cheng Kung University, Tainan 70101, Taiwan; 6 Division of Thoracic Surgery, Department of Surgery, National Cheng Kung University Hospital, College of Medicine, National Cheng Kung University, Tainan 70101, Taiwan; b85401067@gmail.com (Y.-T.Y.); tsengyl@mail.ncku.edu.tw (Y.-L.T.); 7 Department of Medical Research, Ditmanson Medical Foundation Chiayi Christian Hospital, Chiayi 60002, Taiwan; 8 Thoracic Division, Department of Surgery, Ditmanson Medical Foundation Chiayi Christian Hospital, Chiayi 60002, Taiwan; 9 Institute of Molecular Biology, National Chung Cheng University, Chiayi 62102, Taiwan

**Keywords:** lung cancer, c-Met, replication-selective oncolytic adenovirus, rapamycin, autophagy

## Abstract

Lung cancer remains a formidable health challenge due to its high mortality and morbidity rates. Non-small cell lung cancer (NSCLC) constitutes approximately 85% of all lung cancer cases, with small cell lung cancer (SCLC) accounting for the remainder. Both NSCLC and SCLC cells express receptor tyrosine kinases, which may be overexpressed or mutated in lung cancer, leading to increased activation. The c-Met receptor tyrosine kinase, crucial for cell transformation and tumor growth, invasion, and metastasis, became the focus of our study. We used an E1B55KD-deleted, replication-selective oncolytic adenovirus (Ad.What), driven by the c-Met promoter, targeting lung cancer cells with c-Met overexpression, thus sparing normal cells. Previous studies have shown the enhanced antitumor efficacy of oncolytic adenoviruses when combined with chemotherapeutic agents. We explored combining rapamycin, a selective mTOR inhibitor with promising clinical trial outcomes for various cancers, with Ad.What. This combination increased infectivity by augmenting the expression of coxsackievirus and adenovirus receptors and αV integrin on cancer cells and induced autophagy. Our findings suggest that combining a c-Met promoter-driven oncolytic adenovirus with rapamycin could be an effective lung cancer treatment strategy, offering a targeted approach to exploit lung cancer cells’ vulnerabilities, potentially marking a significant advancement in managing this deadly disease.

## 1. Introduction

Lung cancer stands as the leading cause of cancer-related mortality, claiming approximately 1.3 million lives globally each year. It predominantly manifests in two forms: small-cell lung carcinoma (SCLC) and non-small cell lung carcinoma (NSCLC), with the latter further categorized into squamous cell carcinoma, adenocarcinoma, and large cell carcinoma. The genesis of lung cancer is multifactorial, with carcinogens, ionizing radiation, and viral infections being the principal contributors [1,2]. Treatment modalities are tailored based on the cancer’s histological type, extent of dissemination, and the patient’s overall health, with surgery, chemotherapy, and radiation therapy being the standard approaches.

In the early 20th century, the serendipitous observation that patients with malignant tumors showed improvement following severe viral infections or vaccination with attenuated viral vaccines sparked interest in the potential anticancer effects of viruses, marking the inception of viral therapy for tumors [3]. In 1991, the first genetic modification of herpes simplex virus type 1 (HSV-1) involved a thymidine kinase knockout, creating a strain capable of inhibiting malignant brain tumor cells while retaining replication ability, signifying a significant step in oncolytic virus therapy evolution [4]. By 1997, research demonstrated that an adenovirus with attenuated E1B genes, known as ONYX-015, possessed a tumor cell-specific lytic capacity [5]. Following this, in 2005, China approved the use of H101, a recombinant human adenovirus type 5, combined with cytotoxic chemotherapy for nasopharyngeal carcinoma treatment [6]. A significant milestone was reached in 2015 when the U.S. Food and Drug Administration approved T-VEC (Talimogene laherparepvec), a genetically modified HSV-1 encoding the granulocyte-macrophage colony-stimulating factor (GM-CSF) for the treatment of unresectable lesions in melanoma patients post-primary surgery [7]. In 2017, T-VEC’s combination with PD-1 inhibitors in melanoma treatment demonstrated a tumor response rate of 62%, including a 33% complete response rate, heralding a surge in oncolytic virus immunotherapy combination research [8]. In 2021, Teserpaturev/G47Δ (Delytact) was recently conditionally approved in Japan for treating malignant glioma, underscoring the ongoing development and potential of oncolytic virus therapies in oncology [9]. Oncolytic adenoviruses, capable of infecting and lysing cancer cells while sparing normal cells, present a promising avenue for cancer treatment [10]. Their replication within tumor cells not only aids in cell destruction but also amplifies the therapeutic dose locally. The adenovirus serotype 5 is particularly noted for its preferential binding to the coxsackievirus and adenovirus receptor (CAR) on host cells, facilitated by the viral coat protein’s knob domain. Enhancing oncolytic activity through tumor-specific promoter-controlled viral gene expression ensures that viral replication occurs exclusively within cancer cells [11,12,13,14,15,16].

The c-Met proto-oncogene, encoding the c-Met protein, also known as hepatocyte growth factor receptor (HGFR), plays a crucial role in cellular processes [17]. While primarily expressed in epithelial cells, c-Met is also present in endothelial cells, neurons, hepatocytes, hematopoietic cells, and melanocytes. Its activation by the hepatocyte growth factor (HGF) initiates kinase activity leading to transphosphorylation of specific tyrosines, which, in turn, activate various signaling pathways influencing the proliferation, survival, apoptosis, invasion, migration, and angiogenesis of non-small cell lung cancer (NSCLC) cells [18]. Given the relatively infrequent occurrence of c-Met protein overexpression, c-MET gene amplification, and mutations, the sustained activation of Met primarily transpires in a ligand-dependent manner [19]. c-Met protein overexpression is observed in the tissues of NSCLC patients. Conversely, c-Met overexpression typically arises in the context of gene amplification. Elevated c-Met protein expression is more prevalent in cancerous tissues compared with normal tissues, with the extent of overexpression often correlating with the stage and malignancy grade in NSCLC patients. For instance, Tsakonas et al. utilized in situ hybridization and immunohistochemistry to report incidences of Met overexpression and Met amplification at 17% and 2.4%, respectively, among 725 postoperative NSCLC patients. Their analysis revealed heightened c-Met protein expression in NSCLC patients, particularly in adenocarcinoma cases, and established a connection between c-Met protein overexpression and poorer prognosis and lower survival rates in adenocarcinoma patients [20]. Given the intimate association between aberrant Met signaling and the progression of lung cancer, inhibitors targeting Met are presently undergoing preclinical investigations and human trials [21,22].

Rapamycin, a macrolide derived from *Streptomyces hygroscopicus*, is an immunosuppressant primarily employed to thwart organ transplant rejection, particularly in kidney transplants. Its mode of action involves binding to FK-binding protein 12 (FKBP12), thereby forming a complex that inhibits the mammalian target of rapamycin (mTOR) pathway through direct interaction with the mTOR Complex1 (mTORC1) [23]. This property, alongside its anti-proliferative effects, underscores its potential in lung cancer therapy [24,25,26]. Given the heightened presence of c-Met manifestations in lung cancer cells compared with normal cells, the first-generation oncolytic adenovirus, designated ONYX-015, has been re-engineered to be driven under the control of the c-Met promoter. This modification may allow the virus to selectively replicate within c-Met over-expressed lung cancer cells, inducing cell death while minimizing the impact on normal cells. Similar concepts have been applied in our previous studies, using various oncogene promoter-driven oncolytic adenoviruses to combat cancers [13,14,15,16]. To further enhance tumor toxicity, investigation into the combined application of oncolytic adenovirus and rapamycin is warranted, aiming to elucidate any synergistic effects on tumor cell eradication. Furthermore, the molecular mechanisms underlying this synergy were subject to detailed exploration in this study.

## 2. Materials and Methods

### 2.1. Cell Culture and Animal Model

Normal human epithelial cell line SV-HUC-1 [13] and human lung carcinoma cell lines (A549, PC14PE6, CL1-5, CL1-5-F4) and the human embryonic kidney cell line HEK293 were cultured in DMEM supplemented with 10% cosmic calf serum, 2 mmol/L L-glutamine, and 50 µg/mL gentamicin. NOD/LtSZ Prkdc<scid> mice were sourced from the National Cheng Kung University Laboratory Animal Center. These animals were housed under specific pathogen-free conditions, with controlled temperature and lighting. All experimental protocols were approved by the National Cheng Kung University’s Laboratory Animal Care and Use Committee in accordance with the Animal Protection Act of Taiwan (IACUC No. 98085).

### 2.2. Construction of Recombinant Adenoviruses

The adenovirus Ad.TJL, capable of replication and carrying the luciferase (Luc) gene, was constructed by homologous recombination of pShuttle-TJL and pAdEasy-1. The process of constructing pShuttle-TJL is as follows: the E1 gene is cleaved from pAd5YS [13] with *BsrG1* and *Mfe1*, and the same *BsrG1* and *Mfe1* are grafted to the pShuttle to form pShuttle-E1. The E1A promoter was cut from the pGL3-E1A promoter with *HindIII* and *Acc65I* and connected to pcDNA3.1(+) with *HindIII* and *Acc65I* to form the pcDNA3.1(+)-E1A promoter. Then, before the E1A promoter of pcDNA3.1(+)-CMV-Luc, the cmv-Luc was cut from pGL3-CMV-Luc using *BamHI* and NotI and connected to the pcDNA3.1(+)-E1A promoter using *BamHI* and *NotI* in the same way. Finally, the CMV-Luc-E1A promoter on pcDNA3.1(+)-CMV-Luc-E1A promoter was cleaved by *NheI* and the E1 gene of pShuttle-E1 was inserted into the front of the PShuttle-E1 by *SpeI* to form pShuttle-TJL. pShuttle-TJL was then cut into threaded bodies with *PmeI* and fed into BJ5183 bacteria with pAdEasy-1, and the strains homologous reconstituted with pShuttle-TJL and pAdEasy-1 were selected using the antibiotic Kanamycin to obtain pAd-TJL. Finally, pAd-TJL was fed into 293 cells to create Ad.TJL. A replication-competent oncolytic adenovirus, Ad.What, driven by the c-Met promoter, was constructed with similar standard homologous recombination techniques using the plasmid pShuttle-Met and pAdEasy-1. The linear recombinant plasmids cleaved with *PacI* were transfected into 293 cells using lipofectamine 2000 (Invitrogen, ThermoFisher Scientific, Waltham, MA, USA) to generate replication-competent oncolytic adenoviruses. A total of 293 cells were used for virus propagation and titration.

### 2.3. Transcription Activity of c-Met Promoter

Chimeric human c-met promoter (−1093~+60) and CAT reporter plasmid, p1.1 Met-cat, were generously provided by Liu Y [27]. The Met promoter region was subcloned into the pFRL2 plasmid, resulting in p1.1c-Met-Luc. Cells were seeded in 24-well plates and transfected with mixtures containing 1 µg of p1.1c-Met-Luc, 0.8 µg of pTCY-LacZ [28], and 1 µL of liposome in 150 µL Opti-MEM, then incubated overnight at 37 °C in a 5% CO_2_ incubator. After washing with PBS, cells were added with 150 μL dissociation buffer (containing 0.01 M DTT) and placed on ice for 20 min. Centrifuge at 12,000× *g* for 10 min at 4 °C, take 40 μL of the sample and add 20 μL Luciferase Assay Reagent II (Dual-Luciferase^®^ Reporter 1000 Assay System, Promega, Madison, WI, USA) to measure firefly luciferase activity as the target gene expression quantity. After the reaction, add 20 μL Stop & Glo^®^ Reagent (containing Substrate, 50×) (Dual-Luciferase^®^ Reporter 1000 Assay System, Promega) to measure the expression of the CMV promoter-driven renilla luciferase activity as an internal control group.

### 2.4. Immunoblot Analysis

Cell lysates from A549, PC14PE4, CL1-5, CL1-5-F4, and SV-HUC-1 were prepared for immunoblotting against c-Met (Santa Cruz Biotechnology, Dallas, TX), phosphorylated p70S6K (p-p70S6K, Cell Signaling Technology, Danvers, MA, USA), and total p70S6K (Cell Signaling Technology) using corresponding antibodies. Anti-β-actin-peroxidase antibody (Sigma-Aldrich, St. Louis, MO, USA) served as a loading control.

### 2.5. Reverse Transcription Polymerase Chain Reaction (RT-PCR)

A549 cells were treated with 2.5 μg/mL of rapamycin for 1, 3, 6, 12, and 24 h and their RNA was extracted with TRIzol reagents (Invitrogen), and cDNA was synthesized using a Reverse-iT First Strand cDNA synthesis kit (ABgene, ThermoFisher Scientific, Waltham, MA, USA) for RT-PCR with primer pairs specific to c-Met (forward 5′-CCTGACATGGGAGGCAATCTGGG-3′ and reverse 5′-CCTCGTGCTCCTGTTTACCTTGG-3′). The PCR conditions were 30 cycles of 94 °C for 30 s, 52 °C for 30 s, and 72 °C for 1 min.

### 2.6. Determination of Infectability by Adenovirus

The infectability of lung cancer cells by adenovirus was assessed by infecting cells in 24-well plates with Ad.LacZ at varying doses. After 48 h, cells were stained for β-gal activity, and infectability was quantified as the percentage of β-gal-positive cells in representative fields.

### 2.7. Flow Cytometric Analysis

Following rapamycin treatment, A549 cells were labeled with anti-CAR (SantaCruz) and anti-αV integrin antibodies (SantaCruz), followed by FITC-conjugated secondary antibody (KPL). Samples were analyzed using a FACSCalibur flow cytometer. Following Ad.GFP and rapamycin treatments in A549 cells for 24 h, samples were analyzed using a FACSCalibur flow cytometer (BD Biosciences, San Diego, CA, USA). 

### 2.8. Cell Viability and Cytotoxicity Assays

Cell viability in the presence of Ad.What and/or rapamycin and Onyx-015 was evaluated using the WST-1 assay (Abcam, Cambridge, UK) in 96-well plates. The IC_50_ of rapamycin was determined as the concentration, resulting in 50% inhibition of cell viability.

### 2.9. Autophagic Assays

The process of constructing pWPXL-LC3-GFP is as follows: the EGFP-LC3 gene is cleaved from pEGFP-C1-LC3 with *EcoRI* and *AgeI*, and the same *EcoRI* and *AgeI* are grafted to the pMEGA to form pMEGA-EFGP-LC3. The EFGP-LC3 was cut from pMEGA-EFGP-LC3 with *BamH1* and *EcoRI* and connected pWPXL to form pWPXL-LC3-GFP. Cells transfected with pWPXL-LC3-GFP were treated with Ad.What or rapamycin. The autophagic activity was visualized 24 h later by fluorescence microscopy, noting LC3-GFP protein aggregation.

### 2.10. Animal Studies

A549 cells were subcutaneously inoculated into male NOD/LtSZ Prkdc<scid> mice aged 6–8 weeks. Once tumors reached 12–16 mm^3^, mice were treated intratumorally with Ad.What, Ad.LacZ, or saline, and/or orally with rapamycin as per the study design.

### 2.11. Statistical Analysis

Statistical significance was determined using Student’s *t*-test, with *p* < 0.05 considered significant. The type of interaction derived from rapamycin and oncolytic adenovirus in binary combination was described using the median-effect/combination index (CI)-isobologram equation. The CI < 1, =1, and >1 show the combination’s synergism, additive, and antagonism effects. These interactions were analyzed using CalcuSyn software version 2.0 based on the Chou and Talalay method (Biosoft, Cambridge, UK). 

## 3. Results

### 3.1. Proliferation of Oncolytic Adenovirus in the Tumor of Tumor-Bearing Mice

**Figure 1:**
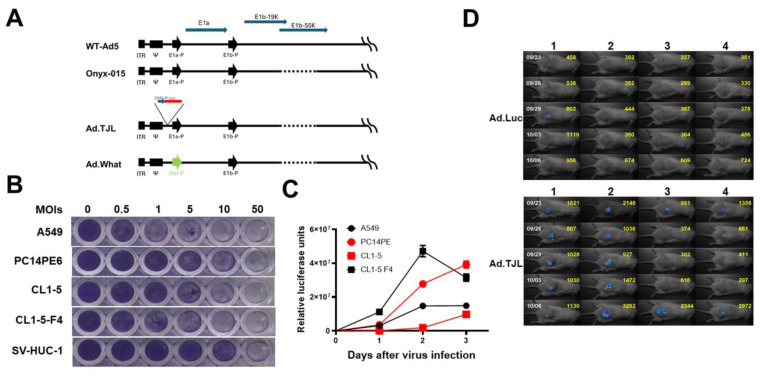
The oncolytic adenovirus, Ad.TJL, demonstrates selective lytic activity against lung cancer cells with minimal impact on normal cells. (**A**) The construction of a series of oncolytic adenoviruses designed to target lung cancer cells specifically. Depiction of the human Ad5 left end with modifications in E1 genes. The wild-type Ad5 with the E1a and E1b genes and their endogenous promoters is shown at the top. The left inverted terminal repeat (ITR), packaging signal (Ψ), the E1a and E1b promoters (E1a-P and E1b-P), and the open reading frames are elucidated. Onyx-015 carries native E1A promoter (E1A-P) to drive E1A expression and an 827-bp deletion with a point mutation to generate a premature stop codon in the E1B55K coding region. Ad.TJL provides a CMV promoter-driven luciferase (CMV-P-Luc) gene to trace the oncolytic virus. Ad.What holds more specific selectivity with the c-Met promoter (Met-P) driven E1a gene instead of the native E1A promoter. (**B**) Different cells were infected with the Ad.TJL virus for 6 h, observed from days 5 to 9 post-infection and subsequently stained with crystal violet to assess viability. (**C**) Cells were infected with the Ad.TJL virus at a multiplicity of infection (MOI) of 1 for 6 h. Cell proteins were harvested post-infection at various time points to detect luciferase activity, providing insights into the virus’s impact on cellular proteins. (**D**) The bioluminescence deriving from Ad.Luc and Ad.TJL in murine tumor models. On day 0, a total of 1 × 10^7^ CL1-5 cells were subcutaneously injected into the backs of NOD/SCID mice. Subsequently, on day 12, 1 × 10^8^ plaque-forming units (PFU) of either Ad.Luc or Ad.TJL were administered directly into the tumors. The detection of bioluminescence was initiated on day 15 and kept observing every three days, utilizing an in vivo bioluminescent imaging detection system, which facilitated the analysis of bioluminescent intensity at the tumor sites, illustrating the efficacy and specificity of Ad.TJL in targeting cancerous cells. Values are the mean ± SD (n = 3).

To investigate the in vivo dynamics of an oncolytic adenovirus, a construct named Ad.TJL was developed. This construct includes a CMV promoter-driven luciferase module alongside Onyx-015, as shown in Figure 1A. Various lung cancer cell lines, including A549, PC14PE6, CL-1-5, and CL1-5 F4, were susceptible to Ad.TJL and exhibit cytopathic effects, as evidenced by crystal violet staining. In contrast, normal human epithelial cell line SV-HUC-1 resisted Ad.TJL infection, as depicted in Figure 1B. Moreover, luciferase activity increased in all lung cancer cell lines tested, indicating that Ad.TJL maintained proliferative activity within these cells (Figure 1C). Notably, unlike Ad.Luc, Ad.TJL demonstrated enhanced bioluminescence signals, specifically in tumor tissues, where bioluminescence from Ad.Luc alone diminished over time (Figure 1D). This highlights the capability of the Ad.TJL oncolytic virus proliferates within cancer cells in vitro and in tumor tissues in vivo, a property not shared by Ad.Luc.

### 3.2. Enhanced c-Met Promoter Activity and Protein Expression Determines Significant Cytopathic Effects of Ad.What in Non-Small Cell Lung Cancer Cells over Normal Cells

**Figure 2:**
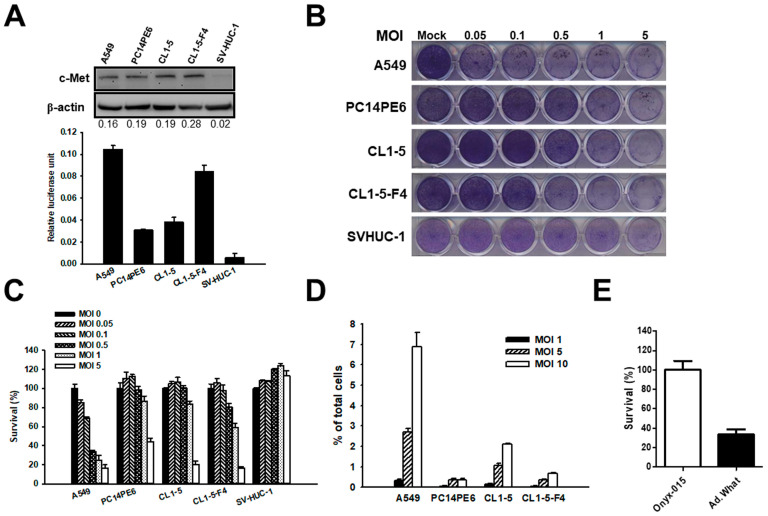
The differential expression and promoter activity of c-Met in lung cancer cells compared with normal epithelial cells, alongside the construction and cytopathic effects of various oncolytic adenoviruses. (**A**) c-Met expression through immunoblotting analysis, with β-actin serving as a quantitative control. The experimental setup involved transient cotransfection of cells with pMet-promoter-Luc and pTCY-LacZ, where luciferase activity was normalized against β-galactosidase activity to assess promoter activity. (**B**) The cytopathic effect (CPE) of Ad.What and the infectivity of adenovirus in both lung cancer cells and normal cells at varying multiplicities of infection (MOIs). Cells were infected with incremental MOIs of Ad.What, with CPE assessed by crystal violet staining between 5 to 9 days post-infection. (**C**) Survival rates, determined by the WST-1 assay, are expressed as a percentage of surviving cells relative to a mock infection control at 4 days post-infection. This comparative analysis provides insights into the selectivity and efficacy of the adenoviral vectors and the c-Met promoter’s role in lung cancer pathogenesis. (**D**) Adenovirus infection rate in lung cancer cells. Cells were infected with Ad.LacZ virus for 6 h and 48 h later were stained with X-gal reagent for intracellular β-galactosidase (β-gal). The infection rate is obtained by dividing the total number of cells stained green by the total number of cells at the time of infection by 100%. Cells were infected with Ad.LacZ at indicated MOIs and incubated for 48 h. Cells were then stained for β-gal expression. Infectability was expressed as a percentage by dividing the number of β-gal-expressing cells by the total number of cells. (**E**) Survival rates, determined by the WST-1 assay in A549 cells, are expressed as a percentage of surviving cells relative to the Onyx-015 infection control at an MOI of 5, four days post-infection. Values are the mean ± SD (n = 3).

To assess c-Met expression in human lung cancer, we performed immunoblot analyses on various human lung adenocarcinoma cell lines, including A549, PC14PE6, CL1-5, and CL1-5-F4, and compared the results to those from normal human epithelial cells. These analyses demonstrated significantly higher c-Met expression in all tested lung cancer cell lines, starkly contrasting to the minimal expression observed in normal cells. Additionally, c-Met promoter activity was notably present in lung cancer cell lines, whereas it was absent in normal cells, as shown in Figure 2A. Leveraging the elevated c-Met expression in cancer cells, we developed an oncolytic adenovirus named Ad.What, which features a c-Met promoter-driven E1a gene, depicted in Figure 1A. In our research on the cytopathic effects of Ad.What, we found that this adenovirus causes significantly higher cytopathic effects in A549 lung cancer cells than in other cancer cell types while exhibiting minimal effects on normal human cells. This differential response suggests a specific interaction between the cytopathic activity of Ad.What and the c-Met expression levels in lung cancer cells, as illustrated in Figure 2B,C. The variation in susceptibility among lung cancer cells to Ad.What-induced cytolysis may also be attributed to differences in adenovirus infectivity, which is further supported by the disparate levels of β-gal expression observed following exposure to Ad.LacZ. This recombinant adenoviral vector, which carries the β-gal reporter gene, serves as evidence of varying infectability among the cell lines tested (Figure 2D). Figure 2E demonstrates that A549 cells infected with Ad.What exhibited significantly reduced viability compared with those infected with Onyx-015, 96 h post-infection.

### 3.3. Enhanced Adenovirus Infectivity and Autophagy by Rapamycin Contribute to Synergistic Effects of Ad.What and Rapamycin in Inducing Cytopathic Effects in Lung Cancer Cells

**Figure 3:**
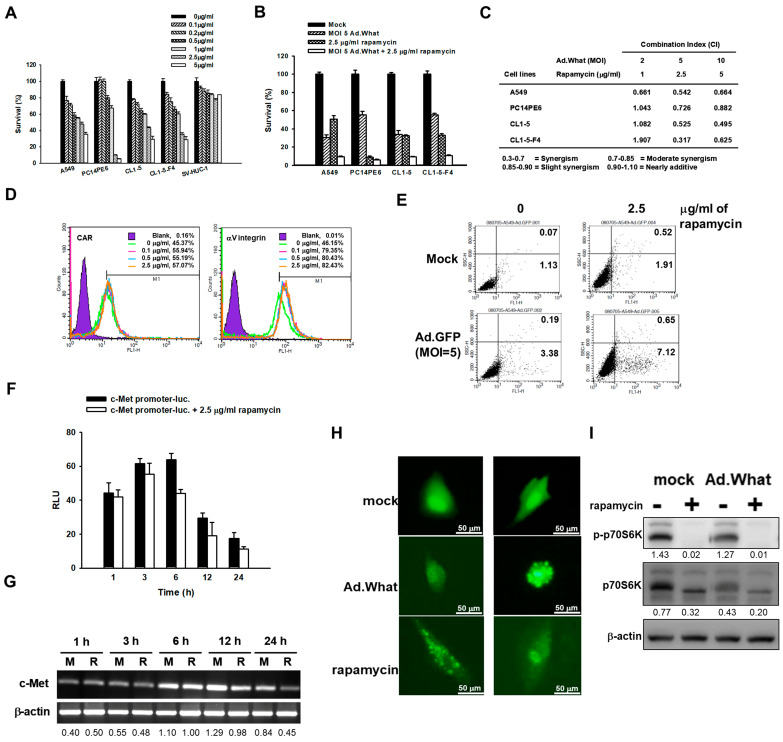
Adenovirus infectivity, autophagy, and synergistic cytopathic effects observed when combining Ad.What and rapamycin treatments on lung cancer cells. (**A**,**B**) Cells were treated with specified concentrations of rapamycin in combination with Ad.What or not. The efficacy of this treatment was evaluated using the WST-1 assay to determine cell survival rates 4 days post-treatment. (**C**) The process for quantifying the synergy between Ad.What and rapamycin. The combination index (CI) was calculated using the CalcuSyn software, which employs the Chou and Talalay method. This calculation aids in assessing the degree of interaction between the two agents, indicating whether their combined effect is synergistic, additive, or antagonistic on lung cancer cell viability. Values are the mean ± SD (n = 3). (**D**) Investigation of how CAR and αV integrin expression levels are affected by rapamycin treatment in A549 cells. The expression levels were measured using flow cytometry 24 h post-treatment, providing insights into the cellular surface receptor dynamics influenced by rapamycin. (**E**) GFP levels in mock-treated, Ad.GFP-infected (MOI of 5) and 2.5 µg/mL rapamycin-treated A549 cells after 24 h were measured using flow cytometry. (**F**) c-Met promoter activity in A549 cells with or without rapamycin treatment at various intervals. The cells were transfected with 1 μg of c-Met-promoter-Luc plasmids using Lipofectamine for 6 h. Subsequently, luciferase activity was detected and normalized to the total protein concentration after 24 h, allowing for an assessment of the promoter’s activity in response to drug treatment. Values are the mean ± SD (n = 3). (**G**) c-Met RNA expression in A549 cells was continuously treated with 2.5 μg/mL of rapamycin at different time points for analysis via RT-PCR. (**H**) Cells were transfected with pWPXL-LC3-GFP to monitor LC3 localization and aggregation, indicating autophagy. Following a 24 h period post-transfection, the cells were treated with an MOI of 5 Ad.What or 2.5 μg/mL of rapamycin. Images documenting the effects of these treatments were captured 24 h later, allowing for visual assessment of autophagic activity and LC3 distribution within the cells. Scale bars represent 50 μm at 1200× magnification. (**I**) Cellular response to Ad.What or mock treatment in the presence of 2.5 mg/mL rapamycin or growth medium control. Cells were treated accordingly, and after 48 h, cell lysates were collected for immunoblotting to assess p-p70S6K and p70S6K levels.

To investigate the cytotoxic impact of rapamycin on both lung cancer and normal cells, we employed the cell proliferation assay at varied concentrations. The findings demonstrated that normal cells displayed considerable resistance to rapamycin when compared with lung cancer cells, as depicted in Figure 3A. We then assessed the combined cytotoxic effects of rapamycin and Ad.What, administered in varying concentrations and at different multiplicities of infections (MOIs), on lung cancer cell lines, illustrated in Figure 3B. Utilizing CalcuSyn software (Biosoft, Cambridge, UK), our analysis aimed to characterize the interaction between these agents as either synergistic, additive, or antagonistic. The results revealed a pronounced synergistic effect between rapamycin and Ad.What on the targeted cells, with combination index (CI) values calculated using the software ranging from 0.3 to 0.7. This effect was especially marked in A549 cells, indicating the highest efficacy of the drug–virus combination. This synergy implies that rapamycin and Ad.What not only act independently to exert cytotoxic effects but also mutually enhance their effectiveness, as illustrated in Figure 3C. 

The lifecycle of an adenovirus within a host cell—ranging from infection replication to the eventual release and cell lysis—underscores the critical phases of its pathogenic process. Here, we investigated the impact of rapamycin on these pivotal stages, particularly focusing on the virus’s ability to infect, replicate, and release from the host cells. Adenoviruses have been known to infect cells primarily via CAR (Coxsackie and adenovirus receptor) and αV integrin cell surface adhesion molecules [29]. Leveraging flow cytometry, we assessed CAR and αV integrin expression levels on the cell surface in response to rapamycin treatment. Our findings reveal that rapamycin significantly upregulates the expression of these cell surface molecules, thereby enhancing the adenovirus’s infectivity (Figure 3D). Additionally, GFP levels were higher in Ad.GFP-infected A549 cells treated with rapamycin compared with those infected with Ad.GFP alone, further confirming that rapamycin can enhance adenovirus infectivity (Figure 3E). Furthermore, we explored the influence of rapamycin on the replication of adenoviruses, particularly through the activity of the E1A promoter, a crucial early gene in the adenoviral replication cycle. The E1A promoter’s function is paramount as it governs the expression of genes essential for subsequent viral replication. In the context of Ad.What, the native E1A promoter has been substituted with the human c-Met promoter, suggesting a potential correlation between intracellular c-Met promoter activity and viral replication. Through reporter gene assays and RT-PCR analysis, we evaluated the activity of the c-Met promoter and the expression of c-Met mRNA in cells treated with rapamycin. Interestingly, our results indicate that rapamycin exerts a minimal effect on the activity of intracellular c-Met activators (Figure 3F,G), suggesting that while rapamycin enhances adenovirus infectivity by upregulating CAR and αV integrin expression, it does not significantly impact c-Met expression.

Previous research has established that rapamycin effectively blocks the mTOR pathway, suppressing intracellular mRNA translation. This suppression adversely impacts the synthesis of cell growth factors, thereby inhibiting cell proliferation. Additionally, since mTOR plays a role in autophagy inhibition, its blockade results in the induction of autophagy within cells [30]. Therefore, our investigation into autophagy, using the autophagy marker LC3-GFP, showed an increase in intracellular green fluorescent LC3 aggregation. This aggregation, observed under the conditions of Ad.What infection or treated with rapamycin, signifies the induction of autophagy by Ad.What (Figure 3H). Furthermore, we employed the immunoblot technique to examine the effects of Ad.What, rapamycin, and their combination on the downstream mTOR molecule, p70S6 kinase (p70S6K), which is crucial for cell growth and metabolism. Our findings reveal that Ad.What significantly diminishes the levels of p70S6K and its phosphorylated form within 48 h of treatment. Moreover, the inhibitory effect on p70S6K was further enhanced when rapamycin was used in conjunction with Ad.What, as demonstrated in Figure 3I. 

### 3.4. Augmenting Antitumor Efficacy with Combined Ad.What and Rapamycin In Vivo

**Figure 4:**
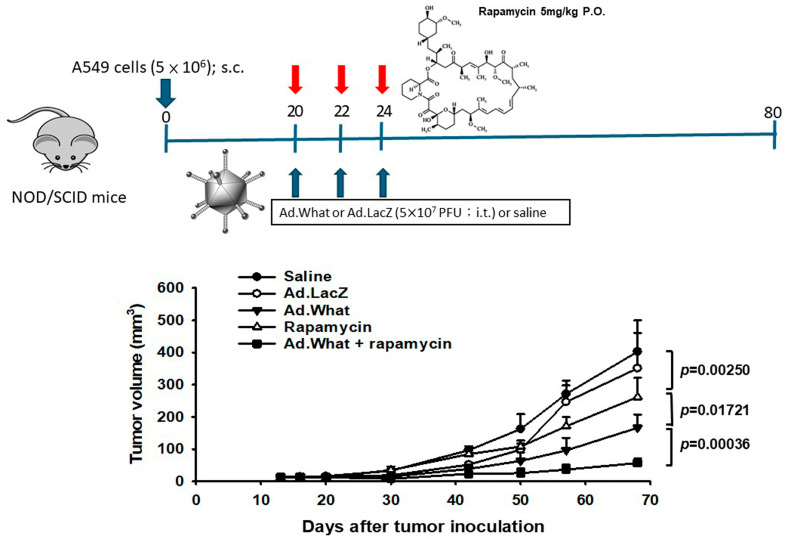
Antitumor effects of Ad.What treatment in combination with rapamycin. Groups of 6–8 male NOD/SCID mice were inoculated s.c. in the flank with A549 cells (5 × 10^6^) at day 0. For single agent treatment, mice received intratumorally (i.t.) with saline, Ad.LacZ or Ad.What (5 × 10^7^ PFU) and orally with rapamycin (5 mg/kg) at days 20, 22 and 24. For combination therapy, mice received i.t. with Ad.What (5 × 10^7^ PFU), followed by oral administration with rapamycin (5 mg/kg) at days 20, 22 and 24. Values are the mean ± SD.

Our animal studies established a subcutaneous tumor model by inoculating NOD/SCID male mice with A549 cells. Treatment commenced on day 20 post-inoculation, assessing the effects of Ad.What and rapamycin, both individually and in combination, on tumor growth. The outcomes demonstrated that while Ad.What and rapamycin each independently curtailed tumor proliferation, their combined application resulted in a significantly more pronounced suppression of tumor growth. This enhanced antitumor effect underscores the potential of leveraging the synergistic capabilities of Ad.What and rapamycin as a more effective therapeutic strategy against tumors, as depicted in Figure 4.

## 4. Discussion

Contrary to earlier beliefs that oncolytic adenoviruses destroy cancer cells without inducing apoptosis [31], recent studies have identified autophagy as the key mechanism behind their effectiveness. The phenomenon, initially documented by Ito et al., demonstrates that autophagy significantly contributes to the adenovirus’s ability to lyse cancer cells, a finding supported by subsequent research, including Alonso et al. and our own observations, which noted increased autophagy markers post-infection [32,33,34,35,36]. Our current study adds to this body of evidence, showing a significant induction in LC3 aggregation in A549 cells. This behavior varies with cell type and is influenced by the viral load, indicating a complex interaction between oncolytic adenoviruses and their target cells through autophagy pathways [35,36,37]. These findings collectively underscore the importance of autophagy in the mechanism of action of oncolytic adenoviruses against cancer cells.

C-MET hyperactivity is crucial in the progression of several cancers, particularly non-small-cell lung carcinoma (NSCLC), by promoting unchecked cell growth. To combat this, c-MET inhibitors, divided into small-molecule tyrosine kinase inhibitors (e.g., crizotinib, tivantinib, cabozantinib, foretinib), monoclonal antibodies (e.g., onartuzumab), and HGF ligand antagonists (e.g., ficlatuzumab, rilotumumab), have been developed [38]. Crizotinib, a notable c-MET inhibitor, is approved for NSCLC patients with ALK gene rearrangements after first-line treatment failure. Additionally, cabozantinib, targeting c-MET and the vascular endothelial growth factor receptor 2, is a second-line treatment for advanced hepatocellular carcinoma (HCC). However, its efficacy is hampered by resistance and low response rates. Research shows that combining cabozantinib with the mTOR inhibitor, rapamycin, effectively counters resistance in HCC by halting cell proliferation, migration, and invasion, marking a significant advance in HCC therapy [39]. Furthermore, when inhibited, the mTOR pathway, a potential target in epithelioid sarcoma treatment, can trigger AKT reactivation, reducing mTOR inhibitors’ effectiveness. This reactivation depends on c-MET signaling, which is highly active in epithelioid sarcoma. Combining mTOR and c-MET inhibitors has demonstrated synergistic effects in reducing tumor growth in preclinical models, offering a new therapeutic strategy for this condition [40]. Overall, the strategic inhibition of c-MET and pathways like mTOR show promise in treating various cancers by overcoming resistance and enhancing therapeutic outcomes. In this study, we targeted the c-MET pathway using the c-MET promoter-driven oncolytic adenovirus, Ad.What, instead of c-MET inhibitors. This kind of replacement may prevent the c-MET inhibitor resistance occurred later. 

The development of oncolytic adenovirus ONYX-015 by Barker and Berk in 1987 represented a pivotal advancement in cancer research, initiating a series of investigations into its tumor-selective replication mechanisms [41]. Initially, the virus was thought to selectively replicate in p53-deficient cancer cells due to the lack of E1B-55K protein, which inhibits p53 activity, a hypothesis posited by Barker and Berk. However, subsequent research, including Rothmann et al.’s 1998 findings, suggested ONYX-015’s tumor specificity might not be exclusively p53-dependent, as it could also replicate in cells with normal p53 function [42,43,44]. The debate continued until 2004, when O’Shea et al. identified that ONYX-015’s tumor specificity was linked to the mechanism of late viral RNA transport within cancer cells rather than p53 presence, underscoring the complexity of viral oncolysis in cancer treatment [45].

In this study, we discovered that rapamycin significantly enhances the expression of CAR and αV cell surface adhesion molecules on A549 cells. Previous research has shown that the histone deacetylase inhibitor FR901228 increases the expression of adhesion molecules on αV integrin-expressing cancer cells and hematopoietic stem cells [46,47]. Moreover, our prior work identified that etoposide could elevate CAR expression in bladder cancer cells [48]. It has also been observed that many drug-resistant cancer cells display an upregulation of αV cell surface adhesion molecules, a trait seen with treatments like Tamoxifen [49] and Cisplatin [50]. Given the tendency for increased expression of αV cell surface adhesion molecules in various drug-resistant cancer cell lines, we hypothesize that A549 cells, when stimulated by rapamycin, might activate a similar yet unidentified mechanism that boosts the expression of αV cell adhesion molecules. This mechanism could confer resistance to rapamycin, whether rapamycin elevates the αV cell surface adhesion molecules on A549 cells via the same pathway observed when other cancer cells develop resistance. This observation could have significant implications for gene therapy, suggesting that the enhanced expression of adhesion molecules on the surface of αV integrin-expressing drug-resistant cancer cells may facilitate adenovirus infection, potentially improving the efficacy of gene therapy approaches.

Our observations in Figure 3 warrant further exploration into the mechanisms driving this synergistic interaction from both the drug and viral perspectives. Interestingly, in PC14PE6 cells known for their high sensitivity to rapamycin, the combination with Ad.What did not yield a synergistic effect. The drug’s efficacy for these cells was significantly lower, and their heightened sensitivity resulted in a limited data range, impeding the software’s ability to generate a CI indicative of synergy. This variance in cell line response underscores the distinct susceptibilities among cell types. Specifically, the acute sensitivity of PC14PE6 cells to rapamycin produces significant effects at lower concentrations, narrowing the range of data observable. This sensitivity complicates the accurate assessment of potential synergistic effects when combining the virus with the drug, suggesting that adjusting the drug concentration may help in identifying a CI that reflects a synergistic interaction. 

## 5. Conclusions

In conclusion, concerning the safety and efficacy of oncolytic adenoviruses, the development of Ad.What, an adenovirus designed with the human c-Met promoter for enhanced safety and tumor targeting, represents a significant advancement. Ad.What inherits ONYX-015’s deletion of the E1B-55K gene and demonstrates selective replication in lung cancer cells with high c-Met activity, sparing normal cells. This specificity, combined with the synergistic effect of co-administering Ad.What with rapamycin, significantly enhances the therapeutic safety and efficacy in lung cancer treatment. Rapamycin amplifies the antitumor effects by upregulating adenovirus receptors and cell adhesion molecules, facilitating viral infection, and inducing autophagy in cancer cells. Although a limitation of our current study is the lack of experimental evidence directly linking the combined treatment’s inhibition of tumor growth in mice to autophagy, this highlights the need for further investigation into the underlying mechanisms. We propose that this synergy allows for the effective targeting of cancer cells with minimized side effects, emphasizing the potential of combining oncolytic adenoviruses and rapamycin as a promising targeted therapy for lung cancer in the future.

## Data Availability

The data supporting the findings of this study are available upon request from the corresponding author.

## Abbreviations

NSCLC: Non-small cell lung cancer, SCLC: small cell lung cancer, Ad.What: E1B55KD-deleted, replication-selective oncolytic adenovirus, driven by the c-Met promoter, Ad.TJL: the oncolytic adenovirus capable of replication and carrying the luciferase (Luc) gene, HSV-1: herpes simplex virus type 1, GM-CSF: granulocyte-macrophage colony-stimulating factor, CAR: coxsackievirus and adenovirus receptor, HGFR: hepatocyte growth factor receptor, mTOR: mammalian target of rapamycin, mTORC1: mTOR Complex1, CI: combination index, MOIs: multiplicities of infection.
